# Factors associated with better emotional, behavioural and educational outcomes in children with mild intellectual difficulties

**DOI:** 10.1002/jcv2.70072

**Published:** 2025-11-25

**Authors:** Foteini Tseliou, Charlotte A. Dennison, Christopher B. Eaton, Jessica M. Armitage, Frances Rice, Stephan Collishaw

**Affiliations:** ^1^ Wolfson Centre for Young People's Mental Health Division of Psychological Medicine and Clinical Neurosciences Cardiff University Cardiff UK; ^2^ Centre for Neuropsychiatric Genetics and Genomics Division of Psychological Medicine and Clinical Neurosciences Cardiff University Cardiff UK

**Keywords:** ALSPAC, education, intellectual difficulties, mental health, positive outcomes

## Abstract

**Background:**

Children with mild intellectual difficulties (MID) are at increased risk of poor mental health and functional outcomes compared to typically developing children. Previous research has primarily focused on deficit‐based comparisons. However, substantial heterogeneity exists in this population, ranging from significant impairment to positive adaptation. Our aim was to test predictors of better emotional, behavioural and educational outcomes in children with MID, with a particular interest in potentially modifiable protective factors.

**Methods:**

Two UK cohorts, the Avon Longitudinal Study of Parents and Children (ALSPAC; *N* = 6926) and the Millennium Cohort Study (MCS; *N* = 8814) were used to examine the associations between independent and cumulative individual, family and social factors in childhood and adolescence and emotional, behavioural and educational outcomes at age 16/17 for children with or without MID. We additionally considered a composite measure of positive developmental outcomes capturing good outcomes across these three domains.

**Results:**

Children with MID (ALSPAC, *N* = 312 [4.5%] and MCS, *N* = 364 [4.1%]) as a group experienced fewer protective predictors compared to children without MID. Physical activity, family social advantage, school enjoyment and good peer relations were each associated with better outcomes in both groups. Cumulative counts of childhood and adolescent factors were strongly associated with better adolescent outcomes, with a ten‐fold difference in the probability of positive outcomes among those with the most and least protective predictors amongst children with MID.

**Conclusion:**

This study underscores the importance of a multi‐faceted approach to supporting children with MID. Further research is required to establish the causal nature of the observed associations, but the findings hold promise for preventative approaches that harness child strengths and build support across family, school and social domains.

## Key points

**What's known?**

- Children with mild intellectual difficulties (MID) are at increased risk of mental health, behavioural and educational outcomes than children without MID.
- Previous research has primarily focused on deficit‐based comparisons between those with and without diagnosed intellectual difficulties, with limited attention to factors associated with positive outcomes in the MID population.

**What's new?**

- Children with MID in both cohorts were less likely to benefit from individual, family and social resource factors such as healthy childhood BMI and physical activity, family social advantage, maternal reading with children, school enjoyment, and positive peer relationships. They were also more likely to experience some risks such as bullying in adolescence.
- Child and adolescent factors predicted adolescent mental health and educational attainment, and there was no evidence that associations differed for those with and without MID.
- A dose‐response relationship was observed, with ten‐fold differences in probability of positive outcomes between children with MID who had the most versus fewest protective factors.

**What's relevant?**

- Children with MID could be better supported through a multi‐faceted approach targeting individual, family, and social domains, with interventions promoting peer relationships being particularly important during adolescence.
- The cumulative nature of protective factors suggests that coordinated, multi‐domain intervention approaches may be more effective than single‐target interventions for improving outcomes in this vulnerable population.

## INTRODUCTION

Cognitive ability exists on a continuum, with those in the lower ranges experiencing increased risk for mental health, behavioural and educational difficulties (Dekker et al., 2002; Einfeld et al., 2011). While formal diagnosis of intellectual difficulties (ID) requires both cognitive limitations (IQ < 70) and significant deficits in adaptive functioning affecting approximately 2%–3% of the population (Totsika et al., 2022), a broader group of children with mild intellectual difficulties (MID) face similar developmental challenges even when they do not meet full diagnostic criteria. Children with cognitive abilities in the borderline to mild impairment range (standardised scores of 50–75) represent approximately 4%–5% of the population the majority of whom are educated in mainstream settings. Growing up, they experience many similar difficulties to those with diagnosed ID, including elevated rates of mental health problems, behavioural difficulties, educational struggles and social exclusion (Fernell & Ek, 2010; Peltopuro et al., 2014). This larger group of children encompassing not only those who might meet ID criteria if comprehensively assessed thus represents an important but understudied population. Our study aims to identify risk and protective factors that account for variation in emotional, behavioural and educational outcomes.

Previous research has primarily focused on comparing outcomes between those with and without diagnosed ID, often finding 2–3 fold increased rates of psychiatric disorders and educational failure (Polanczyk et al., 2015; Shevell et al., 2003). However, this deficit‐focused approach obscures substantial heterogeneity within the MID population, where outcomes range from significant impairment to positive adaptation (Scheffers et al., 2020). Understanding what drives this variation is crucial for developing supportive interventions.

The traditional diagnostic approach to mild intellectual disability, while clinically necessary, inadvertently obscures important variation in functioning and may miss opportunities to understand positive outcomes. Many children with MID demonstrate positive adaptation despite their challenges, yet research has predominantly focused on deficits and diagnostic categorisation rather than examining why some children with similar cognitive profiles thrive while others struggle. This gap is more pronounced for the broader population of children with MID who remain undiagnosed, because they have not undergone comprehensive assessment, because they fall just above the traditional threshold of standardised cognitive ability <70 or because their adaptive functioning does not meet diagnostic thresholds. These children, who may be identified through special educational needs processes or academic struggles rather than clinical pathways, represent a substantial group whose developmental trajectories and support needs remain poorly understood.

The current study examines children with MID (standardised scores 50–75) regardless of adaptive functioning status. This approach has several advantages: First, it captures children who may experience cognitive‐related challenges but lack comprehensive diagnostic assessment, a common situation in population cohorts and real‐world settings (Doody O et al., 2025). Second, it allows examination of the full spectrum of outcomes, including those demonstrating positive adaptation despite cognitive limitations. Third, it provides insights relevant to the larger population of children struggling with cognitive demands in educational and social settings.

Research examining predictors of positive outcomes in various at‐risk populations has identified multiple individual, family, and social factors that promote positive outcomes (Masten, 2007; Rutter, 2006). However, few studies have examined whether these same factors operate similarly for children with MID, or whether different protective processes may be particularly important for this group.

Previous studies of resilience in other high‐risk groups have established well‐replicated associations with more positive outcomes of various individual characteristics and behaviours, including prosocial traits, aspects of positive cognitions (e.g., self‐esteem, an internal locus of control), and healthy lifestyle including frequent physical exercise (Gartland et al., 2019; Luthar et al., 2000; Moore et al., 2023; Scheffers et al., 2020).

Additionally, the family environment is important in supporting positive outcomes for children who experience adversity or individual vulnerability, with strong evidence for beneficial effects of good parental mental health, family socioeconomic advantage, and high‐quality supportive relationships between parents and children (Collishaw et al., 2007; Savage‐McGlynn et al., 2015; Scheffers et al., 2020). Finally, the quality of children's broader social environment is also important for understanding better‐than‐expected outcomes in high‐risk children, especially children's school experiences and the quality of relationships with friends and peers (Cicchetti & Rogosch, 2009; Collishaw et al., 2016; Gartland et al., 2019; Sapouna & Wolke, 2013).

Despite the wealth of evidence on predictors of positive outcomes in various high‐risk populations, only a very small number of studies have considered factors associated with better outcomes in young people with MID. Previous studies have primarily used qualitative and cross‐sectional study designs with small samples or focused on mental health diagnoses as outcomes (Buckley et al., 2020; Einfeld et al., 2011). This represents a significant gap in our understanding, particularly given the heightened vulnerability of this population and the substantial variability in their developmental paths.

### Aims

Study aims were to test (i) the association of MID with emotional, behavioural and educational outcomes in two longitudinal population cohorts; (ii) whether children with or without MID differed in the availability of individual, family and social factors that we hypothesised would be associated with positive outcomes; (iii) whether these predictors were associated with better emotional, behavioural and educational outcomes; (iv) whether these factors behave similarly regardless of cognitive ability level and (v) whether these factors show cumulative effects when considered together. We examine cognitive ability as a continuum and risk factor rather than attempting to diagnose ID, recognising that comprehensive adaptive functioning assessment was not available in these population cohorts.

To extend the generalisability of findings we consider evidence from two longitudinal population cohorts in the U.K., with diverse demographic characteristics from different locations and periods of time. Our focus is on emotional, behavioural and educational outcomes in adolescence (at approximately age 16 years), given heightened vulnerability to mental health problems at this time (Kim‐Cohen et al., 2003), as well as the significance of educational attainment for later life course outcomes (Kosik et al., 2018).

## MATERIALS AND METHODS

This study used two UK longitudinal cohorts, the Avon Longitudinal Study of Parents and Children (ALSPAC) and the Millennium Cohort Study (MCS).

### Samples

The ALSPAC is a population‐based cohort study that recruited pregnant women resident in Avon, UK, with expected delivery dates between 1st April 1991 and 31st December 1992 (Boyd et al., 2013). Enrolled pregnancies were 14,541, with 13,988 children alive at 1 year of age (Fraser et al., 2013). The total sample size for analyses using any data collected after the age of seven is 15,447 pregnancies following further follow‐up (Northstone et al., 2019). Please note that the study website contains details of all the data that is available through a fully searchable data dictionary and variable search tool (http://www.bristol.ac.uk/alspac/researchers/our‐data/). Ethical approval for the study was obtained from the ALSPAC Ethics and Law Committee and the Local Research Ethics Committees.

The MCS follows the lives of 19,517 children born between the 1st of September 2000 and the 11th January 2002 in either England, Scotland, Wales, or Northern Ireland (Connelly & Platt, 2014). During the recruitment process, efforts were made to ensure adequate representation through oversampling of disadvantaged and ethnic minority populations (Plewis et al., 2007). More information can be found at: http://www.cls.ioe.ac.uk/. To account for this selection process, sample design weights were used in the analyses undertaken in the non‐imputed MCS. The data collection for the MCS is approved by the UK National Health Service Research Ethics Committee.

### Mild intellectual difficulties

Mild intellectual difficulties were assessed using cognitive ability tests in middle childhood. In ALSPAC, cognitive ability was assessed at age 8 using Wechsler Intelligence Scale for Children (Wechsler, 2003) to derive a total standardised cognitive ability score. Cognitive ability was assessed in MCS using subsets of the British Ability Scales (BAS) (Brown, 2014). Different subsets were administered at approximately ages 7 and 11 years. We aimed to include one subset assessing non‐verbal and one verbal reasoning abilities; and therefore, utilised the pattern construction (age 7) and verbal similarities (age 11) tests to capture key cognitive domains. The average standardised score across these two tests was taken to derive a standardised cognitive ability score for MCS. While additional BAS subscales were available across waves, we selected these two as they assess fundamental non‐verbal and verbal reasoning abilities that are less dependent on formal academic instruction compared to the BAS attainment‐focused subtests, thereby minimising confounding effects of educational variation.

For each cohort, we transformed raw test scores into *z*‐scores, controlling for age as children taking the cognitive tests in these two cohorts varied in age by several months on each measurement occasion. Standardised scores (mean of 0 and standard deviation of 1) were then grouped into a binary variable identifying a non‐MID group with an estimated standardised cognitive ability above 75 (>−1.67 below the mean) and a MID group with an estimated standardised cognitive ability between 50 and ≤75 (between −1.67 and −3.32 SDs below the mean; ALSPAC = 312 [4.5%] and MCS = 364 [4.1%]). Given our focus on mild ID, we excluded children with severe ID defined as an estimated standardised cognitive ability of <50.

### Child and adolescent predictors

Measures of childhood and adolescent predictors are summarised in Supporting Information S1: Table S1. To enable the construction of cumulative counts (see below) and for ease of interpretation, binary measures were created using validated cut‐points where possible.

#### Prosocial behaviour

Prosocial behaviour was assessed in ALSPAC at ages 8 and 13 years and in MCS at ages 7 and 14 years using a 5‐item subscale of the parent‐rated Strengths and Difficulties Questionnaire (SDQ; Goodman, 1999), which includes questions about sharing readily with other children, and being helpful if someone is hurt/feeling ill (*α* = 0.70; 0.74; Goodman, 2001; Speyer et al., 2023). Each of the five items was parent‐rated on a 3‐point Likert scale (0 = Not true, 1 = Somewhat true, 2 = Certainly true), with a maximum score of 10 and a cut‐off of ≥4 being used to indicate high prosocial traits (Bryant et al., 2020).

#### Physical activity

In ALSPAC being physically active was assessed at age 11 and 13 using accelerometry that captured the average daily minutes of physical activity, for example brisk walking or jogging. Moderate‐to‐vigorous physical activity (MVPA) was derived from the average minutes per day, with MVPA defined as ≥3600 counts per minute based on previous calibrated accelerometry use data (Green et al., 2011). In MCS, physical activity was assessed at age 11 and at 14 using self‐report questions and regular exercise was defined as reporting more than 3 days of exercise per week (Yang, 2019).

#### BMI

BMI, computed as weight (kg) over height (*m*) squared, was also measured at 11 and 13 years in ALSPAC, and 11 and 14 years in MCS, and was adjusted for age. Healthy BMI was defined as a BMI equal to or greater than the 5th percentile and less than the 85th percentile for age (Fiechtner et al., 2017).

#### Locus of control

Locus of control was assessed at 8 years in ALSPAC using the 40 item self‐report Children's Nowicki and Strickland Internal, External Scale (CNSIE; Nowicki & Strickland, 1972) which has been tested across different samples (Beretvas et al., 2007; *a* = 0.68), with individuals grouped as internal or external locus of control.

#### Self‐esteem

Self‐esteem was assessed at 8 years in ALSPAC using the self‐report 6‐item Global Self‐Worth subscale (*a* = 0.67; Guerin & Tatlow‐Golden, 2019) of the Harter's self‐perception profile for Children (Harter, 1985). Scores range from 6 to 24, and those with higher scores indicating greater self‐esteem.

#### Maternal mental health

Self‐reported maternal depression was assessed through the question ‘had depression since the child was born’ at child age 8 months in ALSPAC and the question ‘diagnosed with depression/serious anxiety’ at child age 9 months in MCS.

#### Social advantage

Family social dis/advantage was defined as a household income of below/above 60% of the median for the sample. In ALSPAC (age 10) and MCS (age 11) the family income thresholds used were £240 per week (Weavers et al., 2021) and £440 per week, respectively.

#### Maternal engagement

Maternal engagement in cognitively stimulating activities with the child in the pre‐school period was measured using parent report of how often the mother ‘reads to child’ per week at 3.5 years in ALSPAC, and at 3 years in MCS. Responses were grouped as not often (never/not at all, less often/1–2 per month, ≤1 per week) versus often (nearly/every day, several/3–5 times per week; Shigemasu et al., 2024).

#### School enjoyment

Child school enjoyment was assessed via child‐report in ALSPAC (age 8 and 13) and parent‐report in MCS (ages 7 and 11). Children were grouped as enjoying school (when responding ‘always’ and ‘usually’) versus not (‘sometimes’ and ‘never/not at all’) (Morris et al., 2021).

#### Peer relations

Peer relations were assessed using the parent‐report SDQ (Goodman, 1999) peer relationships five‐item subscale in ALSPAC (8 and 13 years) and in MCS (7 and 14 years), which includes questions such as having at least one good friend and being liked by children (*α* = 0.53; 0.57) (Goodman, 2001; Speyer et al., 2023), with scores ranging from 0 to 10. A cut‐off point of ≥4 was used to indicate good peer relations across timepoints (Bryant et al., 2020).

#### Bullying

Presence or absence of bullying was assessed at 7 and 13 years in ALSPAC and at 7 and 11 years in MCS. It was defined as parent reporting ‘certainly true’ to the question assessing whether the child is ‘being bullied’ (Lereya et al., 2015).

#### Cumulative predictor scores

Two cumulative counts of hypothesised predictors of positive outcomes were created capturing predictors assessed (i) in childhood and (ii) in adolescence. The childhood score included: high prosocial traits, being physically active, healthy BMI, good maternal mental health, family social advantage, mum often reading to child, enjoying school, having good peer relationships and not being bullied. The adolescent score included: high prosocial traits, being physically active, healthy BMI, enjoying school, having good peer relationships and not being bullied. Individuals were grouped as having 0–2, 3, 4, 5 and 6+ predictors (range of 0–9 factors for the childhood score and 0–6 factors for the adolescence score, in both cohorts).

### Adolescent outcomes

Emotional, conduct and educational outcomes were assessed at age 16 years in ALSPAC and age 17 years in MCS.

#### Emotional problems

The parent‐report SDQ emotional subscale (Brann et al., 2018; Goodman, 1999), includes five questions on depressed mood, somatic complaints, general worry, nervousness, and fears each rated as not true, somewhat true, or certainly true (Goodman, 2001; Speyer et al., 2023), and was completed by the child's main carer (usually the child's mother) with scores ranging from 0 to 10 (*α* = 0.71; 0.73). It has been validated as a measure of emotional disorders for individuals aged up to 18 years (Armitage et al., 2023).

#### Conduct problems

The parent‐report SDQ conduct problems subscale contains five questions on temper, disobedience, fighting, lying and stealing (Goodman, 2001; Speyer et al., 2023), with conduct scores ranging 0–10 (*α* = 0.63; 0.67). It has been validated as a measure of symptoms of behavioural disorders in children and adolescents (Goodman et al., 2010).

#### Educational attainment

Educational attainment was assessed as having achieved one or more A*‐C GCSEs for ALSPAC and MCS. In the UK educational system, General Certificate of Secondary Education (GCSE) examinations are typically taken at age 16, with grades ranging from A* (highest) to G (lowest), with A*‐C grades considered passes that meet the benchmark for further education progression.

#### Cross‐domain positive outcomes

Individuals were classified as demonstrating positive outcomes across outcome domains if they: (i) scored in the normal range (≤4) on the SDQ emotional subscale, (ii) in the normal range (≤3) on the SDQ conduct subscale and (iii) achieved one or more A*‐C GCSEs.

### Statistical analysis

Analyses were undertaken using the STATA 18 statistical package.

#### Aims 1 and 2

To address the first aim, we tested the association between childhood MID and emotional, behavioural and educational outcomes at age 16/17. For our second aim, we compared patterns of child and adolescent predictors between young people with and without MID to identify systematic differences in risk and protective factor profiles between groups.

#### Aim 3

Our third aim was to test whether child and adolescent factors are associated with better emotional, behavioural and educational outcomes. We ran univariate linear or logistic regression models separately for those with and without MID to test associations between each child/adolescent predictor and emotional and conduct problem scores and with educational attainment. Univariate models were used to examine the individual effect of each predictor variable separately.

#### Aim 4

Next, to test whether associations between predictors and outcomes differed for young people with and without MID, models were run for the whole cohort and included an interaction term (MID by each predictor). Interaction terms allowed us to test whether the strength or direction of associations varies significantly between groups.

#### Aim 5

Our final aim was to consider whether child and adolescent factors show cumulative effects when considered together. We investigated whether the proportions of children who met criteria for cross‐domain positive outcomes in adolescence (normal range emotional and conduct problems, at least one GCSE pass) varied according to the total count of child or adolescent predictors. By creating composite scores, we aimed to assess the cumulative influence of risk and protective factors. Using logistic regression models, we tested the association between childhood and adolescent cumulative predictor indexes and the probability of young people experiencing a cross‐domain positive adolescent outcome. Analyses were first stratified by MID. We then investigated whether the cumulative indices differed in strength of association with cross‐domain positive outcomes comparing those with and without MID by including the interaction term (MID by cumulative predictor score).

#### Secondary analyses and sensitivity tests

Additional sex‐stratified analyses of associations between MID and each adolescent outcome, and of associations between the cumulative predictor indexes and the indicator of positive outcomes across domains, are presented in the supporting material. Sex stratification was included because developmental trajectories in emotional and behavioural problems in adolescence, and protective factor associations often differ between males and females (Sterba et al., 2007).

Models of associations between predictors and emotional symptoms scores were repeated using the self‐report short Moods and Feelings Questionnaire (Eyre et al., 2021) in ALSPAC and the self‐report SDQ‐E in MCS given that young people may be more accurate informants of their own moods and thoughts.

Additional sensitivity tests presented in online supplementary materials includes analyses repeated using complete‐case unimputed data to test whether imputation procedures influenced the main findings. Analyses using complete‐case data showed broadly similar patterns but with some attenuation of effects, suggesting the need to interpret effect sizes cautiously given the level of missingness.

#### Missing data

Missing data rates for adolescent outcomes varied substantially between cohorts. For example, ALSPAC had 58.5% missing for emotional and conduct problems, while MCS had lower rates of 10.8% for emotional/conduct problems (Supporting Information S1: Table S2). These differences reflect the longitudinal nature of these cohorts, with ALSPAC experiencing greater attrition over its longer follow‐up period. To account for missingness in our variables of interest (see Supporting Information S1: Table S2), we imputed covariates and outcomes using multiple imputation with Fully Conditional Specification in IBM SPSS Statistics Version 27. This approach allows for flexible modelling of different variable types within the same imputation framework, with predictive mean matching used for continuous variables being distribution‐free and not requiring normality assumptions. In total, 50 imputations (10 iterations in each instance) were implemented using logistic regression models for categorical variables and predictive mean matching for continuous variables. We also included auxiliary data to make our missing at random (MAR) assumption more tenable. Given the substantial missing data observed and our assessment that the missingness mechanism was likely MAR, multiple imputation was deemed appropriate to minimise potential bias. Auxiliary variables including birthweight, child ethnicity, child speech problems and reading ability, mother age at birth, mother education and marital status, maternal smoking, and financial difficulties in childhood (Supporting Information S1: Table S3), were added to strengthen the imputation model under the MAR assumption by providing additional information that predicts both missingness patterns and missing values (Madley‐Dowd et al., 2019).

All MCS analyses accounted for the complex survey design, including stratification, clustering and non‐response. This was implemented using Stata’s survey data analysis commands (svyset and svy:) with the appropriate longitudinal survey weights, primary sampling unit, and stratum variables, as per the MCS guidance (Plewis et al., 2007).

### Role of the funding source

The funders of the study had no role in the study design, data collection, data analysis, data interpretation or writing of the report.

## RESULTS

Our ALSPAC analysis sample included 6926 individuals (50.2% female, 94.2% White background and mean standardised cognitive ability score = 104.3 [SD = 16.4]) of whom 4.5% (*n* = 312) had MID, while MCS included 8814 individuals (50.7% female, 85.8% White background and mean standardised cognitive ability score = 105.1[SD = 9.2]) of whom 4.1% (*n* = 364) had MID (Table 1 and Supporting Information S1: Table S4).

**TABLE 1:** Adolescent emotional, behavioural and educational outcomes by MID status in the imputed datasets.

	ALSPAC		*β*/OR (95% CI)	MCS		*β*/OR (95% CI)
	No MID	With MID		No MID	With MID	
	95.5%^a^	4.5%^a^		95.9%^a^	4.1%^a^	
Emotional problems	2.58 (3.21)	3.75 (3.57)	1.06 (0.28 1.85)	3.25 (2.16)	4.01 (2.77)	0.93 (0.56 1.30)
Conduct problems	2.68 (3.10)	3.84 (3.29)	1.17 (0.39 1.96)	2.80 (2.48)	3.93 (3.12)	0.71 (0.42 0.99)
No GCSEs A*‐C	8.6%	38.2%	2.15 (2.10 2.24)	10.0%	32.5%	4.30 (3.27 5.65)
Non‐adaptive	38.1%	68.1%	0.28 (0.19 0.41)	32.1%	61.8%	0.37 (0.30 0.47)

*Note:* Table shows mean (SD) and beta coefficients (95% CI) for emotional and conduct problems mean, and percentages and odds ratios (95% CI) for GCSEs and positive outcomes (adaptive functioning).

Abbreviations: ALSPAC, Avon Longitudinal Study of Parents and Children; GCSE, general certificate of secondary education; MCS, Millennium Cohort Study; MID, mild intellectual difficulties.

^a^ Table 1 shows percentages in the imputed dataset with non‐imputed numbers and percentages corresponding to 6614 (95.5%) without MID and 312 (4.5%) with MID in the ALSPAC cohort and 8450 (95.9%) without MID and 364 (4.1%) with MID in the Millennium cohort. Supporting Information S1: Table S4 presents all outcomes in the non‐imputed data.

### Aim 1: Mild intellectual difficulties and adolescent emotional, behavioural and educational outcomes

In ALSPAC, children with MID had increased parent‐rated emotional problems scores at age 16 (*β* = 1.06 [95% CI 0.28–1.85]), increased parent‐rated conduct problem scores (*β* = 1.17 [0.39–1.96]), a higher proportion not achieving any A*‐C grade GCSE (38.2% vs. 8.6%; OR = 2.15 [2.10–2.24]) and lower rates of cross‐domain positive adaptation (38.1% vs. 68.1%; OR = 0.28 [0.19–0.41]) when compared to children without MID (see Table 2). These findings were replicated in MCS across all outcomes including emotional problems (*β* = 0.93 [0.56–1.30]), conduct problems (*β* = 0.71 [0.42–0.99]), achieving any A*‐C grade GCSE (10.0% vs. 32.5%; OR = 4.30 [3.27–5.65]) and cross‐domain positive adaptation (32.1% vs. 61.8%; OR = 0.37 [0.30–0.47]). Poorer adolescent outcomes for children with MID were observed for both sexes (See Supporting Information S1: Table S5). Thus, despite increased risk of difficulties in the MID group, there was significant variability within this group and around a third exhibited evidence of positive adolescent outcomes.

**TABLE 2:** Patterns of predictors by mild intellectual difficulties (MID) status in the imputed datasets.

	ALSPAC			MCS		
	No MID	With MID	OR (95% CI)	No MID	With MID	OR (95% CI)
Individual						
High prosocial traits (childhood) %	95.4%	92.1%	0.57 (0.32 1.04)	98.5%	97.6%	0.60 (0.32 1.11)
High prosocial traits (adolescence) %	94.4%	91.2%	0.61 (0.33 1.14)	96.0%	93.4%	0.59 (0.39 0.89)
Physically active (childhood) %	77.8%	64.1%	0.51 (0.37 0.70)	55.0%	34.6%	0.43 (0.35 0.53)
Physically active (adolescence) %	62.2%	55.6%	0.76 (0.52 1.12)	71.9%	67.3%	0.80 (0.65 1.00)
Healthy BMI (childhood) %	61.5%	53.5%	0.72 (0.55 0.94)	59.9%	56.1%	0.86 (0.72 0.93)
Healthy BMI % (adolescence)	46.5%	43.9%	0.90 (0.68 1.20)	35.9%	34.8%	0.96 (0.77 1.18)
Internal LoC (childhood) %	60.8%	33.1%	0.32 (0.25 0.41)	NA	NA	NA
High self‐esteem (childhood) %	51.2%	35.5%	0.52 (0.41 0.67)	NA	NA	NA
Family						
Good maternal mental health (childhood) %	85.5%	79.2%	0.65 (0.47 0.89)	76.4%	76.2%	0.99 (0.80 1.22)
Social advantage (childhood) %	50.9%	27.1%	0.36 (0.26 0.48)	34.8%	12.3%	0.26 (0.20 0.34)
Mum often reads to child (childhood) %	89.4%	84.7%	0.66 (0.46 0.95)	79.1%	65.5%	0.50 (0.41 0.62)
School						
Enjoys school (childhood) %	96.6%	92.9%	0.48 (0.25 0.91)	94.2%	89.2%	0.51 (0.38 0.69)
Enjoys school (adolescence) %	88.2%	85.7%	0.80 (0.53 1.23)	92.7%	84.9%	0.44 (0.34 0.58)
Good peer relations (childhood) %	95.2%	87.5%	0.36 (0.23 0.55)	96.2%	91.4%	0.42 (0.30 0.59)
Good peer relations (adolescence) %	94.0	81.7	0.28 (0.19 0.42)	92.4	84.4	0.45 (0.34 0.60)
No bullying (childhood) %	80.7	73.2	0.65 (0.48 0.89)	68.9	67.2	1.00 (0.83 1.23)
No bullying (adolescence) %	79.9	60.1	0.38 (0.28 0.51)	62.1	51.3	0.64 (0.50 0.83)

*Note:* Table shows percentages and odds ratios (95% CI).

Abbreviations: ALSPAC, Avon Longitudinal Study of Parents and Children; BMI, Body Mass Index; LoC, Locus of Control; MCS, Millennium Cohort Study; MID, mild intellectual difficulties; NA: not available.

### Aim 2: Patterns of predictors by mild intellectual difficulties group

In relation to individual factors (Table 2 and Supporting Information S1: Table S6), young people with MID in both cohorts had lower prosocial traits in adolescence, lower rates of physical activity in childhood, and lower rates of healthy BMI in childhood, while in ALSPAC (where additional information on cognitive factors was available) they also had lower self‐esteem and internal locus of control than children without MID.

In terms of family factors, children with MID in ALSPAC were less likely to have mothers reporting good mental health. In both cohorts, children with MID were also less likely to have socio‐economically advantaged parents and mothers that reported often reading to child.

Children with MID in both cohorts were less likely to report that they enjoyed school in childhood. Across childhood and adolescence, there was also a higher proportion of children with MID in both cohorts that did not have good quality peer relationships and who experienced bullying.

### Aim 3: Are predictors associated with emotional, behavioural and educational outcomes?

We then investigated associations between child and adolescent factors and each of the three adolescent outcomes at age 16/17.

#### Emotional problems

**FIGURE 1:**
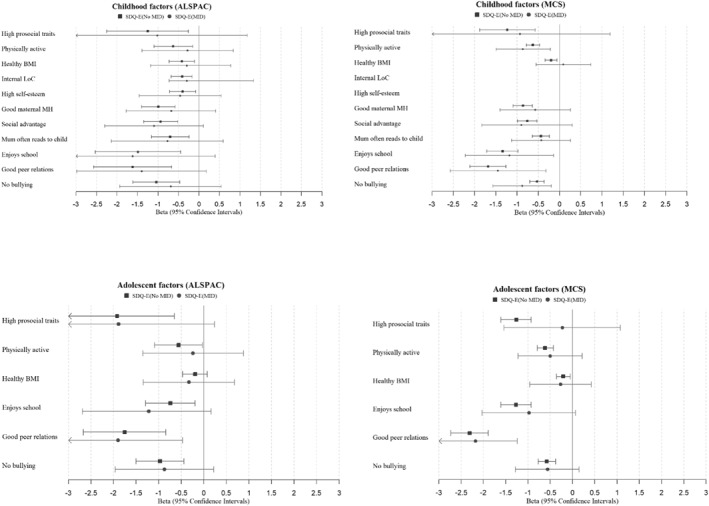
Univariate association of predictors with SDQ emotional problems at age 16/17 for children with or without MID in the imputed datasets. Figure shows beta coefficients (95% CI); no significant interaction between MID and predictor; For beta coefficient, a *β* > 0 means a positive effect, while a *β* < 0 means a negative effect; models adjusted for sex. ALSPAC, Avon Longitudinal Study of Parents and Children; MCS, Millennium Cohort Study; MID, mild intellectual difficulties; SDQ, Strengths and Difficulties Questionnaire.

Figure 1 summarises associations with age 16/17 emotional problems for childhood and adolescent factors, separately for those with or without MID (See Supporting Information S1: Table S7).

For children without MID all childhood factors and almost all adolescent factors (except for healthy BMI) were consistently associated with lower rates of emotional problems across both cohorts. Effect size estimates of predictor associations with emotional problems were of similar magnitude for young people with MID, but with wide confidence intervals. Among those with MID, the only factor associated with emotional problems in both cohorts was adolescent peer relationship quality (ALSPAC *β* = −1.90 [95% CI −3.33 to −0.47]; MCS *β* = −2.18 [95% CI −3.12 to −1.24]). In addition, childhood physical activity (*β* = −0.86 [−1.49 to −0.22]), childhood school enjoyment (*β* = −1.18 [−2.22 to −0.14]), childhood peer relationship quality (*β* = −1.45 [−2.57 to −0.32]) and absence of childhood bullying (*β* = −0.88 [95% CI −1.57 to −0.19]) were associated with lower emotional problems in MCS. Broadly similar patterns were observed when using self‐reported measures of adolescent emotional problems and depression (Supporting Information S1: Table S8).

#### Conduct problems

**FIGURE 2:**
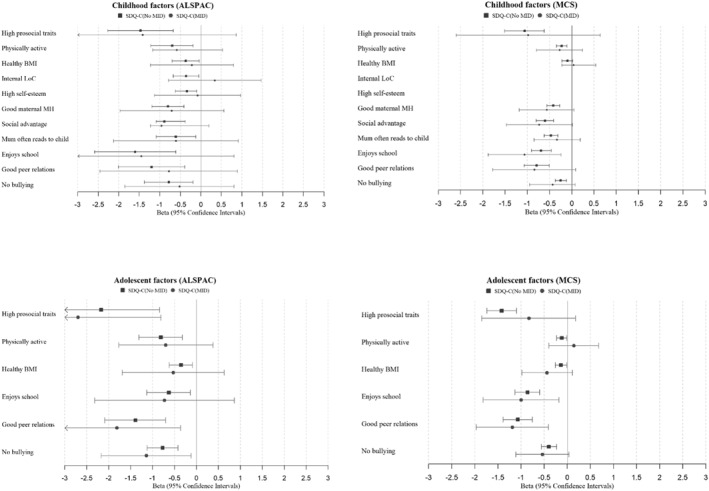
Univariate association of predictors with SDQ conduct problems at age 16/17 for children with or without MID in the imputed datasets. Figure shows beta coefficients (95% CI); no significant interaction between MID and predictor; For beta coefficient, a *β* > 0 means a positive effect, while a *β* < 0 means a negative effect; models adjusted for sex. ALSPAC, Avon Longitudinal Study of Parents and Children; MCS, Millennium Cohort Study; MID, mild intellectual difficulties; SDQ, Strengths and Difficulties Questionnaire.

Figure 2 summarises associations of child and adolescent factors with adolescent conduct problems, separately for those with or without MID (See Supporting Information S1: Table S9).

For children without MID there were associations between most child and adolescent factors and lower conduct problems in both cohorts. Among those with MID, good peer relations was associated with lower conduct problems in both cohorts (ALSPAC *β* = −1.81 [CI −3.25 to −0.36]; MCS *β* = −1.19 [−1.97 to −0.41]). Other predictive associations with child or adolescent factors were not replicated at conventional levels of statistical significance across the two cohorts, but effect size magnitudes were similar between the two groups (see Figure 2).

#### Attainment (attainment of A*‐C GCSEs at age 16)

For children without MID (Supporting Information S1: Table S10), several predictors were robustly associated with educational attainment in both cohorts (physical activity, social advantage, school enjoyment, and adolescent prosocial behaviour, peer relationship quality and absence of bullying). In contrast, for children with MID no robust associations with educational attainment were detected.

### Aim 4: Do predictors of positive outcomes behave differently in those with and without mild intellectual difficulties

Further tests tested for differences in associations of predictors with each outcome between children with and without MID. For all three adolescent outcomes and each predictor, confidence intervals around estimates of association overlapped between children with and without MID, and interaction models revealed no significant interactions by MID group (see Supporting Information S1: Tables S7–S10).

### Aim 5: Are there cumulative effects of predictors on positive outcomes?

**FIGURE 3:**
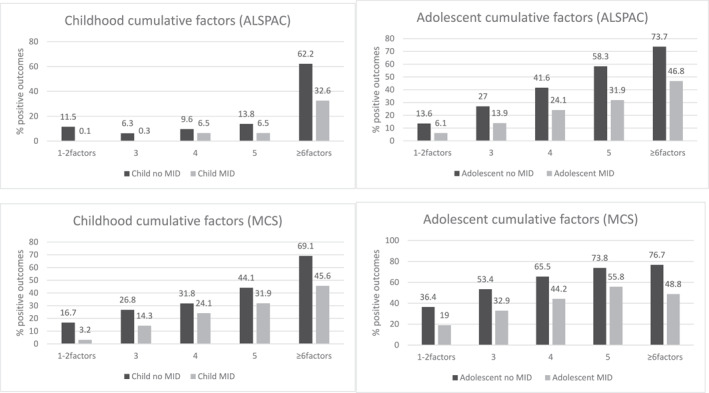
Cumulative effect of childhood and adolescent predictors on positive outcomes in the imputed datasets. Figure shows percentages.

Finally, we investigated cumulative associations of childhood and adolescent factors with positive adolescent outcomes (Figure 3).

Dose response relationships between the number of predictors in childhood or adolescence and positive outcomes were evident in both cohorts (Figure 3). This was true for children with or without MID.

Among young people with MID, a greater number of childhood factors predicted positive outcomes in ALSPAC (OR = 1.29 [CI 1.06–1.57], *p* = .010) with a similar pattern in MCS (OR = 1.79 [0.89–3.55], *p* = .098). The count of adolescent factors predicted positive outcomes for young people with MID in both cohorts (ALSPAC OR = 1.78 [95% CI 1.35–2.53], *p* < .001; MCS OR = 1.45 [95% CI 1.16–1.80], *p* = .001). There were substantial differences in rates of positive outcomes amongst children with MID when comparing those with 6 or more predictors and those with fewer than 3 predictors both for childhood factors (ALSPAC: 32.5% vs. 0.1%; MCS: 45.6% vs. 3.2%) or adolescent factors (ALSPAC: 46.8% vs. 6.1%; MCS: 48.8% vs. 19.0%).

There was no evidence that associations of the cumulative indexes with positive outcomes differed by MID status either for the childhood score (Interactions by MID: ALSPAC OR = 0.72 [−0.28 to 7.16], *p* = .134; MCS OR = 0.47 [−1.15 to 2.09], *p* = .568) or for the adolescent score (Interactions by MID: ALSPAC OR = 0.53 [−0.16 to 1.23], *p* = .131; MCS OR = 0.11 [−1.17 to 1.52], *p* = 0.849).

Similar patterns of findings showing dose response relationships between predictors counts and positive outcomes were observed when stratified by sex (See Supporting Information S1: Figures S1 and S2).

## DISCUSSION

This study investigated individual, family and social factors in childhood and adolescence that may be involved in promoting more positive developmental outcomes for children with MID. The study considered the consistency of findings across two large prospective UK population cohorts. Mild intellectual difficulties even when broadly defined in unselected population cohorts were linked with poorer emotional, behavioural and educational outcomes at age 16/17 when compared young people without MID, in line with previous evidence showing high rates of mental health difficulties in this group (Buckley et al., 2020). Children with MID had less access to many of the supportive factors and resources considered in this study. While individual, family and social factors robustly predicted adolescent emotional, behavioural and educational outcomes in the full cohorts, the pattern was more mixed when focussing specifically on children with MID. However, good quality peer relationships in adolescence showed consistently strong associations with better emotional and conduct outcomes across both groups. Nevertheless, we found no evidence that specific predictors were differentially associated with outcomes in children with MID versus children without MID. When considered together, the predictors considered here appeared to exert cumulative influences on later mental health and educational outcomes with evidence for strong dose response relationships in both cohorts. Rates of positive adaptation across domains amongst children with MID were over 10 times greater in those with 6 or more childhood factors compared to those with fewer than three.

### Factors linked to emotional, behavioural and educational outcomes in children with mild intellectual difficulties

The study extends prior understanding of individual, family and social factors that may be relevant for improving developmental outcomes for children with MID. Most factors showed associations with better emotional, behavioural and/or educational outcomes in adolescence in the full cohort and there was no evidence that these factors were any less important for children with MID versus those without MID. Indeed, there were similar effect size estimates for most predictors we examined, albeit with wide confidence intervals around estimates for the smaller MID group. There was also no evidence that specific factors were of particular (greater) importance for children with MID compared to children without MID.

There was robust support for a link between adolescent peer relationship quality and emotional and behavioural outcomes among children with MID in both cohorts. Previous research has highlighted the importance of good quality social relationships in promoting better outcomes in children and young people exposed to a range of different forms of psychosocial adversity, including children who have experienced maltreatment, bereavement or parent mental illness (Collishaw et al., 2007). The current study highlights similar benefits of good peer relationships for emotional and behavioural outcomes in children with MID. However, positive social experiences were less common in those with MID with 40%–50% experiencing bullying and around three times as many adolescents with MID not experiencing good quality peer relationships. This is a particular concern given that adolescence is a salient period for establishing social bonds and close friendships (Blakemore, 2008) with implications for long‐term social functioning and mental health (Allen et al., 2024). Therefore, it is important to recognise the complex and dynamic nature of the developmental processes by which children develop friendships with past experiences of relationships with peers shaping the social skills and competencies needed to form, maintain and benefit from relationships in future (Allen et al., 2024).

### Positive developmental outcomes

A composite measure of positive outcomes encompassed positive emotional, behavioural and educational outcomes. Previous research has consistently demonstrated that ID are not isolated challenges but manifest across multiple domains of functioning (Emerson & Hatton, 2007). By incorporating emotional and behavioural dimensions alongside educational attainment, we aimed to capture a more holistic view of how children with MID navigate developmental challenges. Emotional problems and conduct difficulties can significantly impact educational engagement, social relationships, and long‐term life outcomes, making them critical components of understanding positive outcomes (Dekker et al., 2002). While children with MID as a group showed poorer outcomes across all three domains, we find evidence for significant variability in outcomes, with around a third of this group showing good adaptation across domains.

### Cumulative effect of predictors

The likelihood of positive outcomes was strongly associated with the number of predictors in childhood and in adolescence. This was the case both for children with MID and those without. The cumulative benefits of child, family and social factors for promoting positive outcomes have been previously investigated in various at risk population groups including children exposed to maltreatment, bereavement or parent depression (Collishaw et al., 2007). Our findings demonstrate a very similar pattern in relation to children with MID.

Children with a greater number of supportive or resource factors were more likely to experience good mental health and educational attainment in adolescence, with a ten‐fold difference in probability of cross‐domain positive outcomes between young people with the greatest and fewest number of protective factors. These findings suggest that similar supportive effects are important for children with and without MID, that there are cumulative effects with information about the number of supportive effects across ecological domains being more important than presence or absence of any specific factor, and that bolstering supportive effects amongst children with MID would likely help improve outcomes for this high risk group.

### Cross‐cohort patterns

The replication across the two cohorts showcased some key points of convergence and divergence. Both cohorts showed nearly identical prevalence of mild intellectual difficulties (MID; 4.5% ALSPAC, 4.1% MCS) with consistently poorer group‐level outcomes, yet approximately one‐third achieved positive adaptation, demonstrating that poor outcomes are not inevitable. Despite methodological differences between cohorts, adolescent peer relationship quality emerged as the most consistent predictor of better emotional and behavioural outcomes for children with MID, underscores the fundamental importance of social connection for this group. The dose‐response relationship between number of protective factors and positive outcomes was very similar across cohorts, with 10‐fold differences between those with most versus fewest protective factors, suggesting that the accumulation of supports matters more than any single intervention target.

In contrast, the broader range of protective factors reaching significance in MCS (e.g., childhood physical activity, school enjoyment for emotional outcomes) compared to ALSPAC may reflect the greater statistical power from lower attrition and more diverse sampling. That similar effect sizes were observed in ALSPAC despite wider confidence intervals suggests these factors may indeed be broadly relevant but require adequate power to detect in the smaller MID subgroups. The availability of cognitive measures (locus of control, self‐esteem) only in ALSPAC revealed additional individual‐level disparities between MID and non‐MID groups, highlighting how cohort‐specific measures can illuminate different aspects of the phenomenon while core patterns remain consistent.

The consistency of these patterns across two cohorts with different characteristics, time periods, and measurement approaches strengthens confidence that these findings reflect genuine phenomena rather than cohort‐specific artifacts. These insights can inform more effective, hope‐oriented approaches to supporting children with MID.

### Strengths and limitations

This study's strengths include replication across two unselected longitudinal population cohorts, the use of child and adolescent assessments, and a consideration of potential promoters of positive adaptation across child, family, and social domains.

Limitations include selective participant dropout, addressed through multiple imputation to limit missing data bias (Madley‐Dowd et al., 2019). However, the high rates of missing outcome data, particularly in ALSPAC, represent a key limitation, suggesting that we cannot exclude the possibility of missingness relating to unmeasured factors associated with poorer outcomes. It is likely that findings present conservative estimates of rates of difficulties in young people with MID given that those with the most severe problems are typically more likely to drop out of studies such as this (Wolke, 2009).

Different measures used across cohorts prevented full replication of findings, with differences in sampling and response rates across the two cohorts (ALSPAC being a more advantaged, regional cohort, while MCS included individuals across the UK and oversampled children from more disadvantaged communities). Additionally, some measures, such as household income, were measured using single question assessments potentially not fully accounting for the multidimensional nature of these constructs or contextual factors that might influence their comparability across households and cohorts.

Primary analyses focused on parent‐reported symptoms. Both parent‐ and self‐reports provide unique valuable information for predicting clinical outcomes (Cohen et al., 2019). Previous research comparing informant reports in young people with ID suggests that differences in mental health between young people with and without ID may be more pronounced for parent than youth reports (Emerson et al., 2023). A similar pattern is seen for other neurodiverse groups. For example, young people with Attention Deficit Hyperactivity Disorder tend to under‐report the severity of symptoms of depression relative to their parents (Fraser et al., 2018). In relation to association with predictors, sensitivity analyses using youth reports of emotional problems or depression were broadly comparable.

Statistical power was limited when stratifying by MID status, resulting in wider confidence intervals for the MID group. The definition of MID relied on available cognitive ability measures rather than comprehensive batteries of assessment of cognitive ability, and findings might not generalise to children diagnosed with intellectual disability in clinical practice (Grigorenko et al., 2020). Though our findings should be interpreted as relating to children with MID at risk for intellectual disability, rather than those with confirmed diagnoses, our broader identification could improve understanding of educational and developmental needs by capturing children who may benefit from additional support regardless of formal diagnostic status.

We also note that our focus is specifically on MID and findings may differ for children with severe ID (standardised cognitive ability <50), who often present with more profound accompanying communication and physical limitations, and are linked to a distinct aetiology (e.g., prominence of genomic syndromes) (Sattler, 2002), meaning that factors promoting positive outcomes may differ between those groups of young people.

Finally, caution is warranted regarding potential causal interpretations as analyses were not corrected for multiple testing, and we were unable to test the direction of association between putative predictive factors and outcomes.

### Implications

Predictors across child, family and social domains collectively influenced positive outcomes in children with MID, mirroring findings in the general population. This suggests that mainstream interventions may benefit children with MID, supporting inclusive approaches to promoting child wellbeing.

The cumulative associations with positive outcomes observed for potentially modifiable factors both in childhood and adolescence are substantial, and the findings hold promise for improving emotional, behavioural and educational outcomes for children with MID.

Our findings highlight the importance of social relationships as an area of increasing vulnerability in the lives of young people with MID as they grow up, in accord with previous studies which have highlighted higher rates of social exclusion, loneliness and peer victimisation (Berchiatti et al., 2022; Richards et al., 2001). What is noteworthy, however, is that there is considerable variability in the social experiences of young people with MID in our study. Indeed, many reported good quality peer relationships, and our findings showed strong associations between better quality peer relationships sand positive developmental outcomes. This points to potential opportunities for intervention. First, it highlights the need to identify difficulties in social relationships early on. Second, it is important to consider barriers to inclusion within mainstream school settings. Third, schools should consider how to tailor anti‐bullying strategies so that they address the higher than average rate of peer victimisation experienced by young people with MID. Finally, it is also important to consider the potential utility of evidence‐based social skills or friendship interventions, but evidence on the efficacy of such interventions in neurodiverse populations remains limited (Cordier, 2023).

Findings also indicate the need for a broad perspective that accounts for cumulative influences of risk and protective factors across individual, family and school domains. This suggests the need to move toward multi‐domain assessment and intervention approaches with coordinated intervention plans that promote strengths and address vulnerabilities across multiple areas of children's lives concurrently. This would recognise that different areas of functioning, relationships, and supports are intertwined and that improvements in one domain (such as parent‐child relationships or friendships) can have cascading positive effects that promote better developmental outcomes in children with neurodevelopmental vulnerabilities (Powell et al., 2021).

Future research should focus on better understanding how multi‐domain interventions can be implemented to maximise their impact on long term outcomes for children with MID. In particular, a better understanding is needed of how to most effectively promote healthier lifestyles for children with MID, provide support for their families, improve parents' own mental health, build supportive friendships, reduce bullying, and how to create enjoyable and engaging learning environments.

## CONCLUSION

Individual, family and social factors together predicted positive emotional, behavioural and educational in young people with MID in both cohorts. There was no evidence that associations between specific predictors and outcomes differed between those with and without MID. However, young people with MID were less likely to experience many resource and support factors. The specific predictor most robustly supported was the quality of adolescent peer relationships which was associated with better emotional and behavioural outcomes for children with MID in both cohorts.

## AUTHOR CONTRIBUTIONS

**Foteini Tseliou**: Formal analysis; conceptualization; methodology; writing—original draft; writing—review and editing; investigation; software. **Charlotte A. Dennison**: Writing—original draft; writing—review and editing; methodology. **Christopher B. Eaton**: Writing—original draft; writing—review and editing; methodology. **Jessica M. Armitage**: Writing—review and editing; writing—original draft; **Frances Rice**: Writing—original draft; writing—review and editing; funding acquisition. **Stephan Collishaw**: Conceptualization; methodology; writing—original draft; writing—review and editing; investigation; funding acquisition.

## CONFLICT OF INTEREST STATEMENT

The authors declare no conflicts of interest.

## ETHICAL CONSIDERATIONS

Informed consent for the use of data collected via questionnaires and clinics was obtained from ALSPAC participants following the recommendations of the ALSPAC Ethics and Law Committee at the time (Bristol and Weston Health Authority: E1808 Children of the Nineties: Avon Longitudinal Study of Pregnancy and Childhood (ALSPAC) [28th November 1989]). Ethical approval was obtained for all waves of the MCS through the National Health Service (NHS) Research Ethics Committee (REC; MCS1 9 months 2000/1 South West MREC MREC/01/6/19). Ethical procedures included informed written parental consent and assent from participants.

## Supporting information

Supporting Information S1

## Data Availability

The data are available upon request and subject to cohort‐specific executive data access procedures. ALSPAC data access is regulated through a system of managed open access. Please note that the ALSPAC study website contains details of all the data that is available through a fully searchable data dictionary and variable search tool (http://www.bristol.ac.uk/alspac/researchers/our‐data/). The University of London Centre for Longitudinal Studies owns the copyright for the MCS data used in this study. The MCS data are held/curated by the UK Data Service. Anyone wishing to use the MCS data (found at: https://discover.ukdataservice.ac.uk/series/?sn=2000031) must register and submit a data request to the UK Data Service at http://ukdataservice.ac.uk/.
