# Supplementary material for: Factors associated with better emotional, behavioural and educational outcomes in children with mild intellectual difficulties
Source: JCPP Adv. 2025 Nov 25;6(3):e70072. doi: 10.1002/jcv2.70072 (PMC13339398; doi:10.1002/jcv2.70072)
Supplement: Supplementary file 1 [file JCV2-6-e70072-s001.docx]

**Factors associated with better emotional, behavioural and educational outcomes in children with mild intellectual difficulties**

**Supporting Information**

Table S1. Measures of childhood and adolescent predictors of positive outcomes in ALSPAC and MCS

|  | ALSPAC | MCS | Childhood | Adolescence |
| --- | --- | --- | --- | --- |
| Individual |  | |  |  |
| High prosocial behaviour | SDQ prosocial subscale (≥4) | SDQ prosocial subscale (≥4) | √ | √ |
| Physical activity | minutes of physical activity (≥3600 counts per minute) | exercise per week (≥3 days) | √ | √ |
| Healthy BMI | eight (kg) over height (m) squared (≥5th & ≤85th percentile for age) | weight (kg) over height (m) squared (≥5th & ≤85th percentile for age) | √ | √ |
| Intern  al locus of control | Children’s Nowicki and Strickland Internal, External Scale (≥ 20) | - | √ |  |
| High self-esteem | Global Self-Worth subscale of Harter’s self-perception profile for Children (≥15) | - | √ |  |
| Family |  | |  |  |
| Good maternal mental health | Did not have depression since the child was born | Was not diagnosed with depression/ serious anxiety | √ |  |
| Social advantage | household income above 60% of the median for the sample (£240 per week) | household income above 60% of the median for the sample (£440 per week) | √ |  |
| Maternal engagement | Mother reads to child (“nearly every day” & “several times per week”) | Mother reads to child (“every day” & “3-5 times per week”) | √ |  |
| Social |  | |  |  |
| School engagement | enjoys school (“always” and “usually”) | enjoys school (“always” and “usually”) | √ | √ |
| Good peer relations | SDQ peer subscale (≥4) | SDQ peer subscale (≥4) | √ | √ |
| No experience of bullying | Not being bullied (“not true”) | Not being bullied (“never”) | √ | √ |

Table S2. Missingness across the two cohorts

| Measures | ALSPAC | MCS |
| --- | --- | --- |
| **Individual** | *6,926* | *8,814* |
| MID (childhood) | 0% | 0% |
| Female | 0% | 0% |
| High prosocial traits (childhood) | 17.4% | 2.4% |
| High prosocial traits (adolescence) | 25.0% | 8.2% |
| Physically active (childhood) | 31.0% | 7.6% |
| Physically active (adolescence) | 48.4% | 6.7% |
| Healthy BMI (childhood) | 19.1% | 3.2% |
| Healthy BMI (adolescence) | 29.3% | 11.4% |
| Internal LoC (childhood) | 6.4% | *NA* |
| High self-esteem (childhood) | 6.6% | *NA* |
| **Family** |  |  |
| Good maternal mental health (childhood) | 10.6% | 0.1% |
| Social advantage (childhood) | 30.7% | 0.2% |
| Mum often reads to child (childhood) | 10.2% | 8.8% |
| **School** |  |  |
| Enjoys school (childhood) | 16.8% | 6.6% |
| Enjoys school (adolescence) | 20.7% | 7.7% |
| Good peer relations (childhood) | 17.4% | 2.5% |
| Good peer relations (adolescence) | 25.0% | 8.2% |
| No bullying (childhood) | 17.1% | 6.7% |
| No bullying (adolescence) | 27.2% | 42.8% |
| **Outcomes** |  |  |
| Emotional problems (adolescence) | 58.5% | 10.8% |
| Conduct problems (adolescence) | 58.5% | 10.8% |
| No GCSEs A*-C (adolescence) | 63.2% | 19.4% |

*Table shows percentages; NA: not available

Table S3. Variables included in multiple imputation

| ALSPAC | MCS |
| --- | --- |
| **Auxiliary variables** | |
| Maternal marital status (32 weeks gestation by carer) | Maternal marital status (9 months by carer) |
| Maternal academic qualification (32 weeks gestation by carer) | Maternal academic qualification (9 months by carer) |
| Ethnicity (32 weeks gestation by carer) | Ethnicity (9 months by carer) |
| Financial problems (8 weeks by carer) | Financial problems (3 years by carer) |
| Birthweight (at delivery by carer) | Birthweight (9 months by carer) |
| Smoking (18 weeks gestation by carer) | Smoking (9 months by carer) |
| Speech problems (4 years by carer) | Speech problems (7 years by carer) |
| Reading ability (7 years in clinic) | Reading ability (7 years by carer) |
| Age of mother (8 weeks by carer) | Age of mother (9 months by carer) |
| **Predictors / Covariates** | |
| Sex (at birth by carer) | Sex (9 months by carer) |
| ADHD (8 years by carer) | ADHD (7 years by carer) |
| ADHD (13 years by carer) | ADHD (14 years by carer) |
| Special education needs (7 years by teacher) | Special education needs (7 years by teacher) |
| Special education needs (11 years by teacher) | Special education needs (11 years by teacher) |
|  |  |
| SDQ prosocial (8 years by carer) | SDQ prosocial (7 years by carer) |
| SDQ prosocial (13 years by carer) | SDQ prosocial (14 years by carer) |
| Physical activity (11 years in clinic) | Physical activity (11 years by carer) |
| Physical activity (13 years in clinic) | Physical activity (14 years by carer) |
| BMI (11 years in clinic) | BMI (11 years by child) |
| BMI (13 years in clinic) | BMI (14 years by child) |
| Locus of control (8 years in clinic) | - |
|  |  |
| Self-esteem (8 years in clinic) | - |
| Maternal mental health (8 months by carer) | Maternal mental health (9 months by carer) |
| Social advantage (10 years by carer) | Social advantage (11 years by carer) |
| Mum reads to child (3 years by carer) | Mum reads to child (3 years by carer) |
| School enjoyment (8 years by child) | School enjoyment (7 years by carer) |
| School enjoyment (13 years by child) | School enjoyment (14 years by carer) |
| SDQ peer (8 years by carer) | SDQ peer (7 years by carer) |
| SDQ peer (13 years by carer) | SDQ peer (14 years by carer) |
| Bullying (7 years by carer) | Bullying (7 years by carer) |
| Bullying (13 years by carer) | Bullying (11 years by carer) |
| **Outcomes** | |
| SDQ Emotional (16 years by carer) | SDQ Emotional (17 years by carer) |
| SDQ Conduct (16 years by carer) | SDQ Conduct (17 years by carer) |
| GCSEs A*-C (17 years by child) | GCSEs A*-C (17 years by child) |

Table S4. Adolescent emotional, behavioural and educational outcomes by mild intellectual difficulties (MID) status in the non-imputed datasets

|  | **ALSPAC** | | | | **MCS** | | | |
| --- | --- | --- | --- | --- | --- | --- | --- | --- |
|  | No MID | No MID  Missingness | With MID | With MID  Missingness | No MID | No MID  Missingness | With MID | With MID  Missingness |
|  | 6,614 (95.5%) |  | 312 (4.5%) |  | 8,450 (95.9%) |  | 364 (4.1%) |  |
| Emotional problems | 1.43 (1.81) | 36.6% | 1.95 (1.96) | 50.0% | 1.94 (2.19) | 29.9% | 2.70 (2.49) | 37.8% |
| Conduct problems | 1.01 (1.35) | 36.5% | 1.43 (1.74) | 49.7% | 1.09 (1.40) | 29.8% | 1.63 (1.76) | 37.6% |
| No GCSEs A*-C | 61 (2.5%) | 62.6% | 19 (28.4%) | 78.5% | 591 (8.7%) | 36.4% | 73 (25.4%) | 47.8% |
| Non-adaptive | 248 (11.7%) | 67.9% | 24 (44.4%) | 81.7% | 1,427 (22.9%) | 42.1% | 100 (43.3%) | 54.8% |

*Table shows mean (SD) emotional and conduct problems mean, and percentages for GCSEs and positive outcomes; Data also present percentages of missingness across outcomes by MID

Table S5. Emotional and educational outcomes by sex in the imputed datasets

|  | ALSPAC | |  | MCS | |  |
| --- | --- | --- | --- | --- | --- | --- |
|  | **Men** | | | | |  |
|  | No MID | With MID | β / OR (95%CI) | No MID | With MID | β / OR (95%CI) |
|  | 95.3% | 4.7% |  | 95.5% | 4.5% |  |
| Emotional problems | 2.69 (3.31) | 3.76 (3.41) | 1.15 (0.23 2.07) | 3.03 (2.95) | 4.01 (3.60) | 0.98 (0.52 1.44) |
| Conduct problems | 2.61 (3.17) | 3.83 (3.68) | 1.13 (0.21 2.04) | 2.81 (2.34) | 3.98 (3.03) | 0.64 (0.30 0.99) |
| No GCSEs A*-C | 10.4% | 40.7% | 2.16 (2.09 2.29) | 12.5% | 37.1% | 4.14 (2.88 5.95) |
| Non-adaptive | 38.6% | 69.7% | 0.27 (0.17 0.42) | 30.9% | 55.9% | 0.35 (0.25 0.49) |
|  | **Women** | | | | |  |
|  | No MID | With MID | β / OR (95%CI) | No MID | With MID | β / OR (95%CI) |
|  | 95.6% | 4.4% |  | 95.4% | 4.6% |  |
| Emotional problems | 2.94 (3.03) | 3.95 (3.13) | 1.00 (0.16 1.84) | 3.66 (2.98) | 4.07 (3.74) | 0.87 (0.41 1.34) |
| Conduct problems | 2.47 (3.12) | 3.68 (3.44) | 1.21 (0.31 2.10) | 2.51 (2.26) | 3.84 (2.80) | 0.77 (0.39 114) |
| No GCSEs A*-C | 6.9% | 35.3% | 2.13 (2.07 2.24) | 7.7% | 28.0% | 4.66 (3.25 6.68) |
| Non-adaptive | 37.6% | 66.3% | 0.30 (0.19 0.48) | 32.8% | 55.5% | 0.39 (0.29 0.53) |

*Table shows mean (SD) & beta coefficients (95%CI) for emotional and conduct problems mean, and percentages and odds ratios (95%CI) for GCSEs and positive outcomes

Table S6. Sociodemographic characteristics across cohorts in the non-imputed datasets

|  | ALSPAC | | MCS | |
| --- | --- | --- | --- | --- |
|  | No MID | With MID | No MID | With MID |
| Individual |  |  |  |  |
| High prosocial traits (childhood) % | 5,322 (96.7%) | 214 (96.4%) | 8,162 (98.7%) | 332 (97.5%) |
| High prosocial traits (adolescence) % | 4,850 (97.1%) | 195 (96.5%) | 7,483 (96.0%) | 302 (93.8%) |
| Physically active (childhood) % | 3,647 (79.1%) | 112 (66.7%) | 4,415 (57.9%) | 119 (35.2%) |
| Physically active (adolescence) % | 2,256 (65.4%) | 68 (58.6%) | 5,712 (72.8%) | 221 (64.0%) |
| Healthy BMI (childhood) % | 3,347 (62.2%) | 122 (55.2%) | 4,968 (62.1%) | 204 (59.7%) |
| Healthy BMI % (adolescence) | 2,223 (47.2%) | 83 (44.6%) | 2,773 (37.3%) | 119 (40.6%) |
| Internal LoC (childhood) % | 3,780 (60.9%) | 91 (32.9%) | *NA* | *NA* |
| High self-esteem (childhood) % | 3,267 (52.8%) | 105 (37.1%) | *NA* | *NA* |
| Family |  |  |  |  |
| Good maternal mental health (childhood) % | 5,104 (86.0%) | 209 (80.6%) | 6,570 (77.6%) | 286 (77.1%) |
| Social advantage (childhood) % | 2,471 (53.8%) | 62 (30.2%) | 3,169 (46.1%) | 49 (20.4%) |
| Mum often reads to child (childhood) % | 5,361 (89.8%) | 216 (85.7%) | 6,275 (83.7%) | 210 (71.8%) |
| School |  |  |  |  |
| Enjoys school (childhood) % | 5,443 (98.0%) | 205 (97.2%) | 7,476 (94.7%) | 295 (89.3%) |
| Enjoys school (adolescence) % | 4,722 (89.3%) | 180 (88.2%) | 7,272 (93.6%) | 298 (86.6%) |
| Good peer relations (childhood) % | 5,283 (90.1%) | 203 (91.0%) | 7,972 (96.6%) | 312 (90.6%) |
| Good peer relations (adolescence) % | 4,768 (95.6%) | 172 (85.2%) | 7,216 (92.8%) | 276 (81.8%) |
| No bullying (childhood) % | 4,575 (83.1%) | 179 (77.2%) | 5,364 (67.6%) | 227 (65.9%) |
| No bullying (adolescence) % | 3,975 (82.0%) | 124 (63.6%) | 3,079 (63.9%) | 96 (55.2%) |

*Table shows numbers and percentages; NA: not available

Table S7. Univariate linear regression models of the association of predictors with SDQ emotional problems at age 16 in the imputed datasets

|  | ALSPAC |  |  | MCS |  |  |
| --- | --- | --- | --- | --- | --- | --- |
|  | No MID | With MID | Interaction | No MID | With MID | Interaction |
| Childhood | | | | | | |
| Individual |  |  |  |  |  |  |
| High prosocial traits | **-1.25 (-2.25 -0.26)** | -1.02 (-3.22 1.18) | 0.27 (-1.55 2.09) | **-1.23 (-1.88 -0.57)** | -0.93 (-3.05 1.19) | 0.29 (-1.69 2.27) |
| Physically active | **-0.63 (-1.10 -0.15)** | -0.28 (-1.39 0.84) | 0.35 (-0.71 1.42) | **-0.63 (-0.78 -0.47)** | **-0.86 (-1.49 -0.22)** | -0.21 (-0.81 0.39) |
| Healthy BMI | **-0.42 (-0.73 -0.11)** | -0.20 (-1.18 0.78) | 0.23 (-0.72 1.18) | **-0.20 (-0.34 -0.06)** | 0.09 (-0.55 0.74) | 0.30 (-0.30 0.89) |
| Internal LoC | **-0.41 (-0.68 -0.17)** | 0.30 (-0.73 1.33) | 0.72 (-0.34 1.77) | *NA* | *NA* | *NA* |
| High self-esteem | **-0.40 (-0.72 -0.08)** | -0.46 (-1.46 0.54) | -0.05 (-0.97 0.86) | *NA* | *NA* | *NA* |
| Family |  |  |  |  |  |  |
| Good maternal mental health | **-0.99 (-1.40 -0.58)** | -0.68 (-1.78 0.41) | 0.30 (-0.73 1.33) | **-0.86 (-1.09 -0.64)** | -0.57 (-1.40 0.26) | 0.29 (-0.46 1.04) |
| Social advantage | **-0.94 (-1.35 -0.52)** | -1.10 (-2.30 0.11) | -0.15 (-1.21 0.90) | **-0.76 (-0.99 -0.53)** | -0.90 (-1.83 0.03) | -0.14 (-1.01 0.72) |
| Mum often reads to child | **-0.70 (-1.16 -0.24)** | -0.77 (-2.14 0.59) | -0.08 (-1.38 1.22) | **-0.44 (-0.64 -0.24)** | -0.43 (-1.13 0.26) | 0.01 (-0.67 0.68) |
| School |  |  |  |  |  |  |
| Enjoys school | **-1.49 (-2.53 -0.45)** | -1.62 (-3.65 0.40) | -0.15 (-1.95 1.65) | **-1.34 (-1.71 -0.98)** | **-1.18 (-2.22 -0.14)** | 0.15 (-0.80 1.11) |
| Good peer relations | **-1.62 (-2.57 -0.67)** | -1.40 (-2.98 0.18) | 0.22 (-1.16 1.60) | **-1.68 (-2.11 -1.26)** | **-1.45 (-2.57 -0.32)** | 0.23 (-0.82 1.28) |
| No bullying | **-1.04 (-1.61 -0.47)** | -0.69 (-1.93 0.54) | 0.33 (-0.67 1.33) | **-0.53 (-0.70 -0.36)** | **-0.88 (-1.57 -0.19)** | -0.35 (-0.98 0.28) |
| Adolescence | | | | | | |
| Individual |  |  |  |  |  |  |
| High prosocial traits | **-1.92 (-3.19 -0.65)** | -1.89 (-4.03 0.24) | 0.02 (-1.72 1.75) | **-1.26 (-1.68 -0.84)** | -0.23 (-1.54 1.07) | 1.03 (-0.20 2.27) |
| Physically active | **-0.56 (-1.09 -0.03)** | -0.24 (-1.35 0.88) | 0.34 (-0.64 1.32) | **-0.62 (-0.79 -0.43)** | -0.50 (-1.22 0.22) | 0.14 (-0.51 0.78) |
| Healthy BMI | -0.19 (-0.47 0.08) | -0.33 (-1.34 0.68) | -0.11 (-1.08 0.86) | **-0.21 (-0.36 -0.05)** | -0.27 (-0.96 0.42) | -0.05 (-0.69 0.60) |
| School |  |  |  |  |  |  |
| Enjoys school | **-0.74 (-1.29 -0.19)** | -1.22 (-2.69 0.16) | -0.48 (-1.79 0.82) | **-1.27 (-1.61 -0.93)** | -0.98 (-2.03 0.07) | 0.28 (-0.67 1.24) |
| Good peer relations | **-1.75 (-2.67 -0.84)** | **-1.90 (-3.33 -0.47)** | -0.16 (-1.30 0.99) | **-2.31 (-2.73 -1.89)** | **-2.18 (-3.12 -1.24)** | 0.12 (-0.76 1.01) |
| No bullying | **-0.97 (-1.50 -0.44)** | -0.87 (-1.96 0.22) | 0.10 (-0.86 1.06) | **-0.58 (-0.77 -0.38)** | -0.56 (-1.28 0.15) | 0.01 (-0.65 0.66) |

*Table shows beta coefficients (95% CI); NA: not available; ^no significant interaction between MID and predictor; For beta coefficient, a *β* > 0 means a positive effect, while a *β* < 0 means a negative effect; models adjusted for sex

Table S8. Univariate linear regression models of the association of predictors with short Moods and Feelings Questionnaire (sMFQ) at age 16

|  | ALSPAC |  |  | MCS |  |  |
| --- | --- | --- | --- | --- | --- | --- |
|  | No MID | With MID | Interaction | No MID | With MID | Interaction |
| Childhood | | | | | | |
| Individual |  |  |  |  |  |  |
| High prosocial traits | -0.52 (-1.65 0.61) | -2.20 (-9.10 4.69) | -1.46 (-7.68 4.76) | -0.09 (-0.67 0.48) | -1.15 (-2.39 0.09) | -1.06 (-2.36 0.25) |
| Physically active | -0.34 (-0.76 0.17) | 0.93 (-2.01 3.87) | 1.27 (-1.25 3.80) | **-0.39 (-0.53 -0.24)** | **-0.80 (-1.49 -0.11)** | -0.39 -1.09 0.32) |
| Healthy BMI | **-0.66 (-1.07 -0.28)** | -1.84 (-4.17 0.48) | -1.19 (-3.26 0.87) | **-0.12 (-0.25 0.01)** | 0.46 (-0.13 1.05) | 0.57 (-0.04 1.17) |
| Internal LoC | **-0.46 (-0.84 -0.09)** | 1.69 (-0.67 4.06) | 2.13 (-0.03 4.30) | *NA* | *NA* | *NA* |
| High self-esteem | **-0.92 (-1.27 -0.56)** | -1.02 (-3.33 1.29) | 0.03 (-2.02 2.08) | *NA* | *NA* | *NA* |
| Family |  |  |  |  |  |  |
| Good maternal mental health | **-1.12 (-1.66 -0.58)** | -1.30 (-4.27 1.67) | -0.33 (3.03 2.37) | **-0.53 (-0.68 -0.39)** | -0.07 (-0.76 0.63) | 0.47 (-0.22 1.16) |
| Social advantage | **-0.66 (-1.05 -0.27)** | 0.11 (-2.35 2.58) | 0.78 (-1.39 2.95) | **-0.26 (-0.39 -0.13)** | -0.16 (-1.11 0.79) | 0.11 (-0.86 1.07) |
| Mum often reads to child | -0.33 (-0.96 0.29) | 0.75 (-2.77 4.27) | 1.07 (-2.18 4.31) | -0.02 (-0.18 0.13) | 0.04 (-0.76 0.83) | 0.06 (-0.71 0.83) |
| School |  |  |  |  |  |  |
| Enjoys school | **-1.93 (-3.28 -0.58)** | -3.12 (-1.0.44 4.21) | -1.23 (-7.54 5.08) | **-0.50 (-0.77 -0.22)** | -0.33 (-1.59 0.94) | 0.17 (-1.14 1.49) |
| Good peer relations | **-1.55 (-2.51 -0.59)** | 1.35 (-2.21 4.91) | 2.86 (-0.47 6.19) | **-0.68 (-1.02 -0.33)** | **-1.53 (-2.61 0.45)** | -0.85 (-1.96 0.26) |
| No bullying | **-1.18 (-1.68 -0.67)** | -1.83 (-4.52 0.86) | -0.67 (-3.13 1.79) | **-0.24 (-0.38 -0.11)** | -0.38 (-1.12 0.35) | -0.14 (-0.88 0.59) |
| Adolescence | | | | | | |
| Individual |  |  |  |  |  |  |
| High prosocial traits | **-1.34 (-2.51 -0.17)** | -1.14 (-9.13 6.85) | 0.02 (-7.48 7.51) | **-0.37 (-0.68 -0.06)** | 0.76 (-0.41 1.93) | 1.13 (-0.07 2.33) |
| Physically active | -0.15 (-0.60 0.30) | 0.74 (-2.59 4.07) | 0.89 (-1.86 3.64) | **-0.55 (-0.71 -0.40)** | 0.38 (-1.05 0.28) | 0.17 (-0.50 0.84) |
| Healthy BMI | -0.41 (-0.79 0.04) | -2.16 (-4.63 0.32) | -1.80 (-3.94 0.33) | -0.09 (-0.22 0.04) | 0.42 (-1.57 1.80) | 0.52 (-0.19 1.22) |
| School |  |  |  |  |  |  |
| Enjoys school | **-1.35 (-1.98 -0.71)** | -2.25 (-6.22 1.72) | -0.87 (-4.37 2.63) | **-0.40 (-0.66 -0.13)** | -0.35 (-1.42 0.71) | 0.03 (-1.02 1.08) |
| Good peer relations | **-1.53 (-2.42 -0.64)** | **-2.69 (-5.94 -0.55)** | -1.19 (-4.38 1.99) | **-0.94 (-1.21 -0.67)** | **-0.94 (-1.78 -0.11)** | -0.01 (-0.87 0.86) |
| No bullying | **-2.33 (-2.81 -1.86)** | -2.23 (-4.54 0.69) | 0.04 (-2.09 2.16) | **-0.41 (-0.58 -0.23)** | -0.99 (-3.22 2.06) | -0.53 (-1.35 0.29) |

*Table shows beta coefficients (95% CI); NA: not available; ^no significant interaction between MID and predictor; For beta coefficient, a *β* > 0 means a positive effect, while a *β* < 0 means a negative effect; models adjusted for sex

Table S9. Univariate linear regression models of the association of predictors with SDQ conduct problems at age 16 in the imputed datasets

|  | ALSPAC |  |  | MCS |  |  |
| --- | --- | --- | --- | --- | --- | --- |
|  | No MID | With MID | Interaction | No MID | With MID | Interaction |
| Childhood | | | | | | |
| Individual |  |  |  |  |  |  |
| High prosocial traits | **-1.47 (-2.27 -0.67)** | -1.42 (-3.70 0.86) | 0.51 (-1.98 2.08) | **-1.06 (-1.51 -0.62)** | -0.98 (-2.60 0.64) | 0.09 (-1.43 1.62) |
| Physically active | **-0.70 (-1.22 -0.19)** | -0.59 (-1.17 0.53) | 0.11 (-1.01 1.23) | **-0.23 (-0.34 -0.11)** | -0.27 (-0.79 0.24) | -0.06 (-0.52 0.40) |
| Healthy BMI | **-0.37 (-0.70 -0.04)** | -0.22 (-1.23 0.80) | 0.14 (-0.84 1.13) | -0.10 (-0.22 0.02) | 0.04 (-0.45 0.54) | 0.13 (-0.30 0.56) |
| Internal LoC | **-0.36 (-0.67 -0.05)** | 0.34 (-0.79 1.47) | 0.70 (-0.47 1.88) | *NA* | *NA* | *NA* |
| High self-esteem | **-0.34 (-0.62 -0.10)** | -0.08 (-1.13 0.97) | 0.28 (-0.68 1.24) | *NA* | *NA* | *NA* |
| Family |  |  |  |  |  |  |
| Good maternal mental health | **-0.80 (-1.19 -0.41)** | -0.71 (-1.97 0.56) | 0.10 (-1.12 1.32) | **-0.42 (-0.56 -0.27)** | -0.56 (-1.18 0.05) | -0.14 (-0.69 0.41) |
| Social advantage | **-0.89 (-1.39 -0.38)** | -0.96 (-1.23 0.20) | -0.07 (-1.13 1.09) | **-0.60 (-0.80 -0.41)** | -0.73 (-1.47 0.01) | -0.11 (-0.76 0.53) |
| Mum often reads to child | **-0.61 (-1.09 -0.12)** | -0.61 (-2.13 0.91) | -0.01 (-1.40 1.39) | **-0.47 (-0.62 -0.31)** | -0.33 (-0.85 0.19) | 0.14 (-0.36 0.65) |
| School |  |  |  |  |  |  |
| Enjoys school | **-1.60 (-2.59 -0.61)** | -1.45 (-3.72 0.81) | 0.16 (-1.92 2.24) | **-0.69 (-0.91 -0.46)** | **-1.06 (-1.88 -0.24)** | -0.36 (-1.10 0.37) |
| Good peer relations | **-1.20 (-2.01 -0.39)** | -0.78 (-2.46 0.89) | 0.42 (-1.07 1.91) | **-0.79 (-1.07 -0.51)** | -0.84 (-1.78 0.09) | -0.05 (-0.90 0.80) |
| No bullying | **-0.78 (-1.38 -0.19)** | -0.52 (-1.85 0.81) | 0.27 (-0.84 1.38) | **-0.25 (-0.37 -0.12)** | -0.43 (-0.95 0.08) | -0.19 (-0.65 0.28) |
| Adolescence | | | | | | |
| Individual |  |  |  |  |  |  |
| High prosocial traits | **-2.17 (-3.50 -0.84)** | **-2.70 (-4.60 -0.81)** | -0.52 (-2.23 1.97) | **-1.42 (-1.74 -1.10)** | -0.83 (-1.85 0.18) | 0.59 (-0.35 1.52) |
| Physically active | **-0.81 (-1.31 -0.32)** | 0.70 (-1.77 0.38) | 0.12 (-0.86 1.09) | **-0.12 (-0.23 -0.01)** | 0.14 (-0.40 0.68) | 0.22 (-0.27 0.71) |
| Healthy BMI | **-0.35 (-0.62 -0.09)** | -0.53 (-1.69 0.63) | -0.19 (-1.26 0.89) | **-0.14 (-0.26 -0.01)** | -0.44 (-0.98 0.11) | -0.31 (0.79 0.17) |
| School |  |  |  |  |  |  |
| Enjoys school | **-0.63 (-1.13 -0.14)** | -0.73 (-2.32 0.86) | -0.10 (-1.61 1.42) | **-0.86 (-1.13 -0.60)** | **-1.00 (-1.82 -0.18)** | -0.13 (-0.81 0.56) |
| Good peer relations | **-1.39 (-2.09 -0.70)** | **-1.81 (-3.25 -0.36)** | -0.41 (-1.74 0.93) | **-1.07 (-1.39 -0.76)** | **-1.19 (-1.97 -0.41)** | -0.11 (-0.81 0.58) |
| No bullying | **-0.77 (-1.12 -0.42)** | **-1.14 (-2.17 -0.12)** | -0.37 (-1.33 0.59) | **-0.40 (-0.56 -0.23)** | -0.54 (-1.11 0.04) | -0.12 (-0.61 0.37) |

*Table shows beta coefficients (95% CI); NA: not available; ^no significant interaction between MID and predictor; For beta coefficient, a *β* > 0 means a positive effect, while a *β* < 0 means a negative effect; models adjusted for sex

Table S10. Univariate binary logistic regression models of the association of predictors with educational attainment at age 16 in the imputed datasets

|  | ALSPAC |  |  | MCS |  |  |
| --- | --- | --- | --- | --- | --- | --- |
|  | No MID | With MID | Interaction | No MID | With MID | Interaction |
| Childhood | | | | | | |
| Individual |  |  |  |  |  |  |
| High prosocial traits | **2.18 (1.13 4.19)** | 0.90 (0.17 4.76) | 0.42 (0.08 2.09) | 1.45 (0.33 1.75) | 1.85 (0.14 5.21) | 1.34 (0.21 8.36) |
| Physically active | **1.53 (1.07 2.19)** | 1.32 (0.68 2.54) | 0.87 (0.45 1.67) | **1.73 (1.60 1.88)** | 1.81 (0.49 1.33) | 1.09 (0.65 1.81) |
| Healthy BMI | 1.18 (0.80 1.73) | 0.89 (0.43 1.84) | 0.78 (0.40 1.53) | 1.00 (0.85 1.18) | 1.09 (0.67 1.77) | 1.08 (0.66 1.76) |
| Internal LoC | **2.01 (1.44 2.79)** | 1.33 (0.66 2.68) | 0.66 (0.33 1.34) | *NA* | *NA* | *NA* |
| High self-esteem | **1.84 (1.16 2.91)** | 1.93 (0.92 4.06) | 1.06 (0.53 2.12) | *NA* | *NA* | *NA* |
| Family |  |  |  |  |  |  |
| Good maternal mental health | 1.50 (0.95 2.37) | 1.11 (0.49 2.48) | 0.73 (0.32 1.69) | 1.38 (0.68 1.51) | 1.50 (0.50 1.73) | 1.02 (0.59 1.75) |
| Social advantage | **2.44 (1.66 3.58)** | 1.69 (0.79 3.62) | 0.70 (0.30 1.60) | **1.47 (1.39 1.59)** | **1.32 (1.12 1.84)** | 0.68 (0.25 1.83) |
| Mum often reads to child | **2.19 (1.37 3.52)** | 2.38 (0.95 5.96) | 1.08 (0.41 2.82) | **1.64 (1.52 1.78)** | 1.72 (0.44 1.91) | 1.14 (0.68 1.90) |
| School |  |  |  |  |  |  |
| Enjoys school | **2.38 (1.08 5.25)** | 1.78 (0.42 7.60) | 1.58 (0.54 2.73) | **1.47 (1.35 1.64)** | 1.49 (0.41 2.16) | 1.98 (0.86 4.56) |
| Good peer relations | 1.64 (0.82 3.29) | 0.64 (0.20 1.99) | 0.39 (0.13 1.14) | **1.58 (1.41 1.83)** | 0.87 (0.36 2.09) | 1.50 (0.60 3.80) |
| No bullying | 1.09 (0.64 1.84) | 0.71 (0.33 1.50) | 0.64 (0.28 1.45) | 0.86 (0.72 1.03) | 1.02 (0.58 1.77) | 1.18 (0.68 2.05) |
| Adolescence | | | | | | |
| Individual |  |  |  |  |  |  |
| High prosocial traits | **3.52 (1.73 7.19)** | 1.88 (0.48 7.34) | 0.52 (0.16 1.77) | **2.55 (1.37 2.82)** | 0.84 (0.32 2.18) | 1.54 (0.57 4.16) |
| Physically active | **2.12 (1.37 3.30)** | 1.45 (0.69 3.03) | 0.69 (0.33 1.46) | **1.79 (1.66 1.95)** | 1.00 (0.58 1.72) | 1.22 (0.71 2.09) |
| Healthy BMI | 1.35 (0.94 1.92) | 1.13 (0.59 2.15) | 0.87 (0.44 1.72) | 0.96 (0.81 1.14) | 0.91 (0.57 1.46) | 0.93 (0.57 1.52) |
| School |  |  |  |  |  |  |
| Enjoys school | **2.11 (1.30 3.44)** | 2.02 (0.72 5.62) | 0.94 (0.36 2.46) | **1.41 (1.31 1.55)** | 1.49 (0.23 1.86) | 1.21 (0.56 2.65) |
| Good peer relations | **2.79 (1.58 4.93)** | 2.11 (0.85 5.26) | 0.75 (0.32 1.78) | **1.51 (1.39 1.66)** | 1.17 (0.41 1.42) | 1.52 (0.81 2.88) |
| No bullying | **2.03 (1.38 2.98)** | 1.90 (0.98 3.70) | 0.94 (0.49 1.79) | **1.74 (1.59 1.91)** | 1.09 (0.47 1.33) | 1.09 (0.64 1.85) |

*Table shows odds ratios (95% CI); NA: not available; ^significant interaction between MID and predictor; models adjusted for sex

Child cumulative (ALSPAC) men

% positive outcomes

|  | Child no MID | Child MID |
| --- | --- | --- |
| 1-2factors | 0.2 | 0.1 |
| 3 | 8.8 | 0.2 |
| 4 | 11.6 | 0.4 |
| 5 | 13 | 7.1 |
| ≥6factors | 59.3 | 28.9 |

Child cumulative (MCS) men

% positive outcomes

|  | Child no MID | Child MID |
| --- | --- | --- |
| 1-2factors | 22.2 | 3.2 |
| 3 | 25.4 | 17.7 |
| 4 | 32.3 | 25.4 |
| 5 | 44.3 | 31.8 |
| ≥6factors | 69.4 | 44.6 |

Adolescent cumulative (ALSPAC) men

% positive outcomes

|  | Adolescent no MID | Adolescent MID |
| --- | --- | --- |
| 1-2factors | 11.3 | 3.1 |
| 3 | 24.1 | 10.5 |
| 4 | 41.3 | 14.8 |
| 5 | 54.5 | 27.6 |
| ≥6factors | 71.6 | 45.6 |

Adolescent cumulative (MCS) men

% positive outcomes

|  | Adolescent no MID | Adolescent MID |
| --- | --- | --- |
| 1-2factors | 34.1 | 20.4 |
| 3 | 52.6 | 32 |
| 4 | 64.7 | 40.7 |
| 5 | 74.1 | 55 |
| ≥6factors | 76.9 | 50.7 |

Figure S1. Cumulative influences of childhood and adolescent predictors on positive outcomes among men. Data represent percentages in the imputed datasets

Among men with MID, a greater number of adolescent predictors were linked to positive outcomes in ALSPAC (MID adolescent OR: 2.06 (95%CI 1.79 2.38), p<0.001) and MCS (MID adolescent OR: 1.49 (95%CI 1.09 2.03), p=0.012). However, that was not the case for childhood cumulative predictors (ALSPAC MID child OR: 0.89 (0.55 1.23), p=0.122; MCS MID child OR: 1.82 (0.63 2.27), p=0.267).

Child cumulative (ALSPAC) women

% positive outcomes

|  | Child no MID | Child MID |
| --- | --- | --- |
| 1-2factors | 23.3 | 0.1 |
| 3 | 4.3 | 0.4 |
| 4 | 4.7 | 5.8 |
| 5 | 13.4 | 4 |
| ≥6factors | 61.6 | 33.3 |

Child cumulative (MCS) women

% positive outcomes

|  | Child no MID | Child MID |
| --- | --- | --- |
| 1-2factors | 8.9 | 0.2 |
| 3 | 14.9 | 0.5 |
| 4 | 28.2 | 23 |
| 5 | 43.1 | 31.6 |
| ≥6factors | 67.8 | 45.6 |

Adolescent cumulative (ALSPAC) women

% positive outcomes

|  | Adolescent no MID | Adolescent MID |
| --- | --- | --- |
| 1-2factors | 15.1 | 7.1 |
| 3 | 27.8 | 16.6 |
| 4 | 38.2 | 26 |
| 5 | 58.1 | 34.3 |
| ≥6factors | 72.4 | 45.7 |

Adolescent cumulative (MCS) women

% positive outcomes

|  | Adolescent no MID | Adolescent MID |
| --- | --- | --- |
| 1-2factors | 36.8 | 14.6 |
| 3 | 52.8 | 33.2 |
| 4 | 65 | 47.5 |
| 5 | 72.7 | 55.4 |
| ≥6factors | 75.3 | 40.8 |

Figure S2. Cumulative influences of childhood and adolescent predictors on positive outcomes among women. Data represent percentages in the imputed datasets

Among women with MID, a greater number of adolescent predictors were linked to positive outcomes in ALSPAC (MID adolescent OR: 1.98 (95%CI 1.70 2.30), p=0.001) and MCS (MID adolescent OR: 1.42 (95%CI 1.04 1.94), p=0.025). However, that was also the case for childhood cumulative predictors for ALSPAC (MID child OR: 1.62 (1.00 2.37), p=0.012) but not MCS (MID child OR: 1.89 (0.61 3.87), p=0.266).
